# Wetting and Spreading Behaviors of Impacting Metal Droplet Regulated by 2D Ultrasonic Field

**DOI:** 10.1002/advs.202415138

**Published:** 2025-01-30

**Authors:** Yuzhu Zhao, Shijing Zhang, Jing Li, Jie Deng, Yingxiang Liu

**Affiliations:** ^1^ State Key Laboratory of Robotics and System Harbin Institute of Technology Harbin 150001 China

**Keywords:** additive manufacturing, adhesive strength, metal droplet, ultrasonic field, wettability

## Abstract

The wetting and spreading behaviors of metal droplets on solid substrates are critical aspects of additive manufacturing. However, the inherent characteristics of metal droplets, including high surface tension, elevated viscosity, and extreme temperatures, pose significant challenges for wetting and spreading on nonwetting substrates. Herein, this work proposes a strategy that employs a two‐dimensional (2D) orthogonal ultrasonic field to construct a vibration deposition substrate with radial vibration amplitude gradient, thereby enhancing the wettability and adhesive strength of impacting metal droplets ejected by a piezoelectric micro‐jet device. First, a 2D ultrasonic vibration device is designed based on the combination of longitudinal vibration modes. Additionally, oblique and circular vibration trajectories are synthesized. The vibration amplitude distributions and trajectories of the deposition substrate are verified utilizing the finite element method. Subsequently, the experimental results demonstrate that the contact angle is decreased by 24.7%, the spreading diameter is increased by 10.3%, and the adhesive strength is enhanced by 5.4 times compared to deposition on a static substrate. The 2D ultrasonic field facilitates the transition of metal droplets from a non‐wetting state to a wetting state on the nonwetting substrate, which highlights the versatility and adaptability of ultrasonic strategy for expanding the applications of metal droplets.

## Graphical abstract

A strategy is proposed that employs a 2D orthogonal ultrasonic field to enhance the wettability and adhesive strength of impacting metal droplet deposition on a nonwetting substrate. The dynamic behaviors and solidified morphology are precisely regulated by adjusting the ultrasonic intensity. The proposed strategy has significant potential to broaden the application scope of metal droplets within the realm of additive manufacturing.

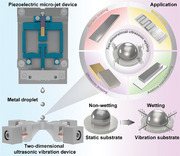

## 1 Introduction

The contact wetting and spreading behaviors at the solid‐liquid interface refer that a liquid expands and spreads across a solid surface. This phenomenon has broad applicability in biological materials,^[^ ^1^, ^2^ ^]^ microfluidic technology,^[^ ^3^, ^4^ ^]^ and ink printing,^[^ ^5^, ^6^ ^]^ etc. Wettability is typically characterized by the contact angle between the solid phase and the liquid phase. A smaller contact angle indicates better wettability.^[^ ^7^, ^8^ ^]^ Strong wettability signifies that a liquid can completely spread and achieve a small contact angle. The wetting and spreading behaviors are influenced by numerous parameters associated with both the solid phase and liquid phase, such as the surface energy and microstructure of the solid and the surface tension of the liquid.

In recent years, the regulation of wettability at the solid‐liquid interface has emerged as a significant area of study. Considerable approaches have been explored to regulate wettability, which can be broadly divided into two categories: Internal strategies and external strategies. The former internal strategies mainly include surface energy and microstructure. On the one hand, the surface energy plays a crucial role in determining the intensity of the interaction. Higher surface energy corresponds to a stronger interaction between the solid phase and the liquid phase.^[^ ^9^ ^]^ Consequently, a solid surface with elevated surface energy is more likely to be wetted, facilitating the occurrence of the wetting phenomenon. On the other hand, the microstructure of the solid surface directly influences the solid‐liquid contact area, which is another crucial factor in determining interface wettability. With ongoing advancements in micro‐nano processing technology,^[^ ^10^, ^11^, ^12^ ^]^ the controllable fabrication of microstructures has become feasible. Surfaces with specific microstructure can effectively reduce the solid‐liquid contact area and enhance surface wettability. Research into the super‐wettability of various biological surfaces, combined with micro‐nano fabrication technologies, has facilitated the imitation of the structure and function of biologically specialized wettability surfaces.^[^ ^13^, ^14^, ^15^ ^]^

The external strategies are those that utilize external energy to disrupt the inherent balance between the solid phase and the liquid phase. As a result, the wettability under specific conditions can be modified by applying external fields such as light,^[^ ^16^ ^]^ electricity,^[^ ^17^, ^18^ ^]^ magnetism,^[^ ^19^, ^20^ ^]^ heat,^[^ ^21^ ^]^ and pressure.^[^ ^22^, ^23^ ^]^ The intervention of external fields serves as an additional factor affecting wettability, allowing for coupled control of this property. A notable characteristic is the capacity to undergo a reversible transition between two distinct wetting states. These methods for controlling wettability above are particularly applicable to low surface tension liquids.

Metal droplets exhibit significant potential for applications in additive manufacturing,^[^ ^24^ ^]^ flexible electronics,^[^ ^25^, ^26^ ^]^ and soft robotics,^[^ ^27^, ^28^ ^]^ attributed to their unique physical properties, which include high electrical conductivity,^[^ ^29^ ^]^ excellent thermal conductivity,^[^ ^30^ ^]^ and remarkable deformability.^[^ ^31^ ^]^ Therefore, regulating the wetting, spreading, and adhering behaviors of metal droplets on solid substrates has emerged as a critical factor for broadening these fields. However, the metal droplets are distinctly characterized by high surface tension, high viscosity, temperature sensitivity, and a tendency to oxidize. The contact‐wetting characteristics and spreading behaviors of metal droplets markedly differ from those of conventional liquids.^[^ ^32^, ^33^, ^34^ ^]^ As a result, achieving effective regulation over the wettability of metal droplets on non‐wetting substrates poses a significant technical challenge, especially when the metal droplets exhibit a certain initial impacting velocity. Achieving good wettability and high adhesion strength is the key technology for broadening the following applications. In particular, controlling the wettability of metal droplets and improving the adhesive strength on non‐wetting substrates can enable surface metallization and coating of non‐wetting materials such as glass, ceramics, and metal matrix composites. In addition, the related technology can be applied to the brazing of metal parts and provide guidance for optimizing technologies such as metal circuits and additive manufacturing based on metal droplet deposition.

The existing methods for enhancing the wettability and adhesive strength of metal droplets primarily include managing substrate temperature,^[^ ^35^ ^]^ incorporating active elements,^[^ ^36^, ^37^, ^38^ ^]^ electrowetting,^[^ ^39^, ^40^, ^41^ ^]^ and employing a one‐dimensional (1D) ultrasonic field.^[^ ^42^, ^43^, ^44^ ^]^ Raising the temperature of the substrate leads to a reduction in the contact angle and an improvement in interfacial adhesive strength. Nevertheless, the wetting behavior of the droplet is affected by the deformation of the substrate. The application of active elements to molten metal is commonly utilized to improve wettability. However, a major limitation is the unavoidable contamination and modification of the physical properties. Electrowetting has been extensively applied to the solidification process of molten metal droplets. However, the setup required for applying the electric field is rather intricate, and the options for substrate materials are restricted. Additionally, numerous studies have been conducted to demonstrate the wetting and spreading behaviors of droplets under the action of ultrasound. The application of high‐intensity ultrasound can indeed enhance the wettability of static droplets on the solid substrate. However, in practical applications such as droplet‐based 3D printing, metal droplets exhibit a specific initial velocity prior to their contact with the solid substrate. Therefore, an innovative strategy based on ultrasound is required to enhance the wettability and adhesive strength of impacting metal droplets on the non‐wetting substrate.

To address the challenges posed by the wetting and spreading behaviors of high‐temperature, high‐viscosity, and high‐surface‐tension metal droplets on substrates that are difficult to wet, in this work, we present a 2D ultrasonic strategy that could effectively enhance the wettability and adhesive strength of metal droplets on nonwetting substrates. The primary innovations and contributions of this work can be summarized as follows: i) A 2D ultrasonic vibration device is developed based on the combination of longitudinal vibration modes. The method for generating a 2D ultrasonic field and the influence of electrical parameters on the vibration characteristics are analyzed. Based on the 2D ultrasonic field, two typical vibration trajectories are planned: oblique vibration and circular vibration; ii) We employ the 2D orthogonal ultrasonic field to construct a vibration deposition substrate with radial vibration amplitude gradient, which is utilized as the energy transfer medium. The radial amplitude gradient can produce radial spreading force within metal droplets, thereby promoting the wetting, spreading, and adhering behaviors of metal droplets on nonwetting substrate; iii) Based on the substrate with 2D vibration trajectory, the wetting and spreading behaviors of metal droplets are precisely regulated by adjusting the ultrasonic intensities. The contact angle, spreading diameter, and adhesive strength of the solidified metal bumps are regulated under different ultrasonic intensities. In summary, the 2D ultrasonic strategy can enhance the wettability and adhesive strength of impacting metal droplets on nonwetting substrate. The implementation of the 2D ultrasonic field enables the transition of metal droplets from a non‐wetting state to a wetting state on nonwetting substrate. These results highlight the versatility and adaptability of the ultrasonic strategy for expanding the application of metal droplets.

## 2 Results and Discussions

### 2.1 Setup for Generation and Deposition of Metal Droplets

**Figure 1:**
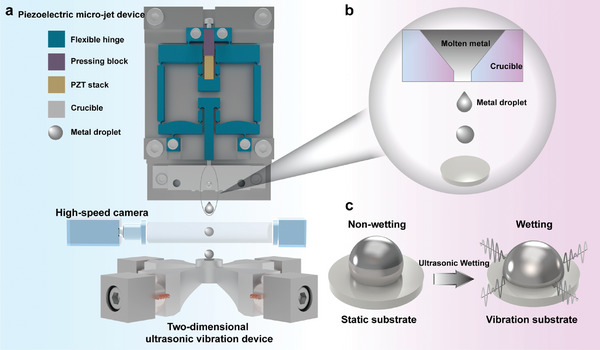
The experimental setup for the generation and deposition of metal droplets. a) Experimental setup based on the piezoelectric micro‐jet device and the 2D ultrasonic vibration deposition device. b) Generation of the metal droplet. c) The wettability of the metal droplet on the static substrate and vibration substrate.

In this work, we focus on the behaviors of impacting metal droplets, specifically examining the wetting, spreading, and adhering characteristics on nonwetting substrates. The overall experimental setup consists of a piezoelectric micro‐jet device and a 2D ultrasonic vibration deposition device, as illustrated in **Figure** **1**a. The piezoelectric micro‐jet device is employed to generate molten metal droplets and comprises several components, including a pretensioned bolt, a pressing block, a lead zirconium titanate (PZT) stack, a flexible hinge, a crucible, and a nozzle. The mechanism for generating metal droplets is as follows: Tin particles with a purity of 99% are introduced into the hot melt cavity through the feed port, where resistance heating is utilized to heat tin particles into a molten state. The trapezoidal excitation signal is modulated by the signal generator and subsequently amplified by the power amplifier before being applied to the PZT stack. The PZT stack generates linear displacement through the inverse piezoelectric effect, which is then amplified and transmitted to the hot melt cavity via the symmetrical flexible hinge mechanism. The volume of the hot melt cavity changes periodically when the PZT stack generates deformation. The volume variations of the cavity influence the molten metals, disrupting the balance of surface tension, liquid static pressure, and flow resistance. Consequently, the molten metal is extruded from the nozzle, leading to the formation of metal droplets, as shown in Figure 1b. To maintain a consistent initial temperature for the metal droplets, a closed‐loop transfer function model of the heating system is established, and a parameter optimization method is utilized to determine the PID controller parameters for effective temperature regulation. In the experiments, the temperature is set to 548 K, with a superheat temperature of 50 K, ensuring that the initial state of the metal droplets is completely molten. By controlling the waveform parameters and voltage amplitude of the excitation signal shown in Figure S1 (Supporting Information), both the size and initial speed of the metal droplets can be adjusted. Following their exit from the nozzle and a brief acceleration phase, the impacting metal droplets wet and spread on the solid deposition substrate, resulting in the formation of metal bumps (Movie S1, Supporting Information).

A 2D ultrasonic vibration device is designed that utilizes piezoelectric ceramics as the driving core, with smooth glass serving as the deposition substrate adhered to the vibration surface. It is well known that most molten metals do not wet the glass.^[^ ^45^ ^]^ In the absence of sinusoidal excitation signals, the glass substrate remains in a stationary state, exemplifying a typical nonwetting system. The vibration device generates 2D horizontal orthogonal ultrasonic fields when two excitation signals are applied, causing the glass deposition substrate to generate horizontal orthogonal micrometers at an ultrasonic frequency. The vibration amplitude and the vibration trajectory of the substrate can be adjusted by varying the voltage amplitude and phase difference of the two‐phase signals, as shown in Figure S2 (Supporting Information). This work proposes a novel strategy to regulate the deposition behaviors of vertically impacting metal droplets by employing 2D horizontal orthogonal ultrasonic fields. The 2D ultrasonic field is transmitted to the interior of the metal droplets through the vibrating substrate. The introduction of 2D ultrasonic fields influences the metal droplets, affecting both the dynamic deposition process and the final solidified morphology, as shown in Figure 1c.

### 2.2 Generation of 2D Orthogonal Ultrasonic field

#### 2.2.1 Structure and Electrical Design

**Figure 2:**
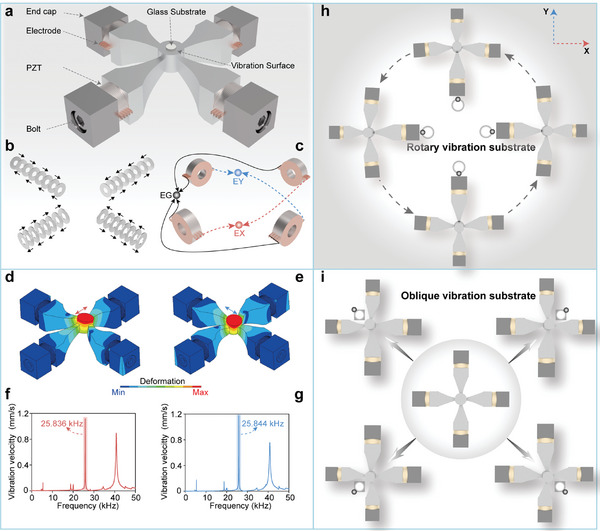
Generation of the 2D ultrasonic field. a) The 3D model and main components of the 2D ultrasonic vibration device. b) Polarization of the PZT elements. c) Electrical configuration. d, e) Second‐order longitudinal modes along X‐axis and Y‐axis. f, g) Vibration characterization tests of the working modes. h) Generation of circular vibration trajectory. i) Generation of oblique vibration trajectory.

**Figure** **2**a illustrates the overall structure of the 2D ultrasonic vibration deposition device, which includes piezoelectric ceramics, electrode sheets, horns, end caps, and bolts. The four longitudinal vibration transducers are connected in parallel, with the central junction of the four branches forming a vibration deposition surface. The exponential horn in each transducer amplifies the deformation of the piezoelectric ceramics, thereby enhancing the vibration amplitude of the deposition surface. To efficiently transmit the multidirectional vibrations generated by the piezoelectric ceramics to the deposition surface, the cross beam and the cylindrical deposition surface are integrally processed utilizing computerized‐numerical‐control machining technology. The vibration device is designed with a sandwich structure, where pre‐tightening force is applied via bolts to ensure a secure connection among the end caps, piezoelectric ceramics, and horns. To facilitate synchronous high‐frequency vibration, we utilize a thin layer of epoxy resin to guarantee high‐strength bonding between the smooth glass substrate and the vibration surface.

The polarization direction and electrical configuration of the piezoelectric ceramics are depicted in Figure 2b,c, respectively. Each driving unit comprises eight pieces of piezoelectric ceramics. All piezoelectric ceramics in the same transducer are polarized along the thickness direction to stimulate longitudinal vibration. Importantly, the adjacent piezoelectric ceramics within each driving unit exhibit opposite polarization directions. When a sinusoidal exciting signal is applied, the piezoelectric ceramics produce longitudinal stretching and shrinkage deformation due to the d_33_ working mode. Therefore, two high‐frequency sinusoidal signals are employed to excite the second‐order longitudinal vibration modes in two horizontal orthogonal directions, which allows the central vibration surface to produce translational vibration along the X and Y directions. As a result, the 2D ultrasonic field can be generated at the glass substrate.

#### 2.2.2 Working Principle

The second‐order longitudinal vibration modes in two orthogonal directions are selected as the working modes, as depicted in Figure 2d,e. We employ the working principle of modal recombination to simultaneously stimulate the two working modes at the same frequency, thereby producing a 2D ultrasonic field. The basis of modal recombination is to achieve frequency degeneracy between the two working modes. Due to the structural symmetry of the vibration device, the frequencies of the two second‐order longitudinal vibration modes are inherently similar. Additionally, a Doppler laser vibrometer is employed to measure the vibrations of the substrate induced by the 2D ultrasonic vibration device in Figure S3 (Supporting Information). The corresponding frequencies of the two working modes are obtained as 25.836 and 25.844 kHz, respectively, resulting in a tiny frequency difference of 8 Hz, which facilitates the effective generation of the 2D ultrasonic field, as shown in Figure 2f,g.

The specific control method of the 2D ultrasonic field is as follows: Under a fixed working frequency, the 2D vibration trajectory of the substrate is modified by adjusting the phase difference of the two excitation signals. Additionally, the vibration intensity is regulated by varying the voltage amplitude. (Note S1, Supporting Information) We utilize the timing of the excitation signals and the resultant mechanical deformation in a single cycle to illustrate the generation of a typical 2D vibration trajectory. Based on the 2D orthogonal ultrasonic fields, we can synthesize two typical vibration trajectories: oblique vibration trajectory and circular vibration trajectory. (Movie S2 and Figure S4, Supporting Information) When the phase difference between the two‐phase excitation signals is 90°, we can identify four characteristic time points in a single cycle to analyze the deformation. The four driving units experience alternating longitudinal deformation due to the phase difference. Therefore, the deformation direction of the vibration surface aligns with either the X‐axis or the Y‐axis at any given characteristic time point. Consequently, a synthesized circular vibration trajectory is formed by the four characteristic points in a single cycle, as illustrated in Figure 2h. When the two excitation signals are identical, exhibiting a phase difference of 0°, the driving units in the X and Y directions deform simultaneously, generating two linear displacement components in orthogonal directions. The combination of the two motion components results in oblique displacements, leading to the substrate exhibiting a high‐frequency reciprocating oblique vibration trajectory, as illustrated in Figure 2i. Furthermore, the resulting vibration trajectory becomes elliptical when the phase difference between the two excitation signals ranges from 0° to 90°, as shown in Figure S5 (Supporting Information).

### 2.3 Vibration Trajectory and Amplitude Distribution of Deposition Substrate

**Figure 3:**
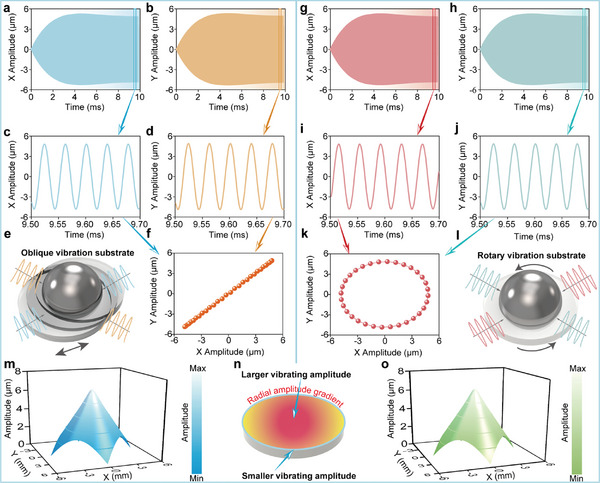
Vibration trajectory and amplitude distribution of substrate. a–d) 2D displacement response of oblique vibration. e) Interaction between the metal droplet and linear vibration substrate. f) Synthesis of oblique vibration trajectory. g–j) 2D displacement response of rotary vibration. k) Synthesis of circular vibration trajectory. l) Interaction between the metal droplet and rotary vibration substrate. m) Vibration amplitude distribution of oblique vibration. n) Illustration of radial amplitude gradient of the substrate. o) Vibration amplitude distribution of circular vibration.

The vibration device generates 2D ultrasonic fields that propagate through the substrate to the metal droplets. The glass substrate serves as a medium for the transmission of the 2D ultrasonic field. Therefore, the response distribution and vibration characteristics of the substrate directly influence the dynamic behaviors of the metal droplets. We employ the finite element method to simulate and analyze the vibration response of the deposition substrate, thereby elucidating the effects of electrical factors on the vibration characteristics. Two types of transient simulation analyses were conducted. The frequency of the two excitation signals was set to 25.84 kHz, the voltage amplitude was 40 V_p‐p_, and the phase differences were 0° and 90°, respectively. The center particle of the deposition substrate was identified through the transient simulation. The vibration displacement response curves in two orthogonal directions are presented in **Figure** **3**a,b,g,h. The vibration displacement response curve of the substrate is categorized into two stages: the transient stage and the steady‐state stage. Subsequently, the sinusoidal curve of each response is extracted for five periods following the attainment of steady state, as illustrated in Figure 3c,d,i,j.

The simulation results indicate that the phase difference of the steady‐state sinusoidal displacement responses in the two orthogonal directions directly corresponds to the phase difference of the input excitation signals. Specifically, the X and Y sinusoidal displacement responses are in phase when the phase difference is 0°; conversely, the X and Y sinusoidal displacement responses are also 90° apart when the phase difference is 90°. Based on the orthogonal steady‐state displacement responses, the synthetic steady‐state vibration trajectory of the substrate is illustrated in Figure 3f,k, respectively. A phase difference of 0° signifies an oblique vibration trajectory, while a phase difference of 90° corresponds to a circular vibration trajectory. Furthermore, the schematic diagram illustrating the interaction between the vibrating substrate and the metal droplets is presented in Figure 3e,l. Additionally, the voltage amplitude is adjusted to evaluate the vibration response of the deposition substrate when the frequency and phase of the excitation signals are constant. It is observed that the vibration amplitude of the substrate increases with an increase in the excitation voltage amplitude, while the vibration trajectory remains unchanged, as shown in Figure S6 (Supporting Information).

Additionally, the vibration response curves from multiple points on the vibrating substrate were analyzed, leading to the creation of a vibration amplitude distribution cloud diagram for the entire substrate, as shown in Figure 3m,o. It was observed that when the substrate vibrates in both oblique and circular manners, a distinct feature emerges: the vibration amplitude is greatest at the center point and diminishes toward the edge point, indicating a radial gradient in vibration amplitude, as illustrated in Figure 3n. Subsequently, the radial amplitude gradient results in a spreading force directed from the center to the edge of the droplet when the vibrating substrate interacts with the metal droplet, thereby enhancing the wettability and adhesive strength.

### 2.4 Wetting and Spreading Behaviors of Impacting Metal Droplet

The prototype is fabricated, and a series of experiments are conducted with metal droplet deposition on the glass substrate. A high‐speed camera is utilized to capture the wetting and spreading behaviors of the metal droplets. The effective interaction time between the ultrasonic field and the metal droplet occurs before the droplet is completely solidified. To maximize the effective interaction time and ensure the durability and repeatability of wettability and adhesion strength, we maintain that the duration of ultrasound application in all the experiments is no less than 3 s. We examine the dynamic processes of metal droplets on three different types of substrates: a static substrate, an oblique vibrating substrate, and a rotary vibrating substrate. By comparing the dynamic behaviors of metal droplets on the stationary substrate with those on the other two vibrating substrates, the mechanism of the 2D ultrasonic field on metal droplet dynamics can be revealed.

**Figure 4:**
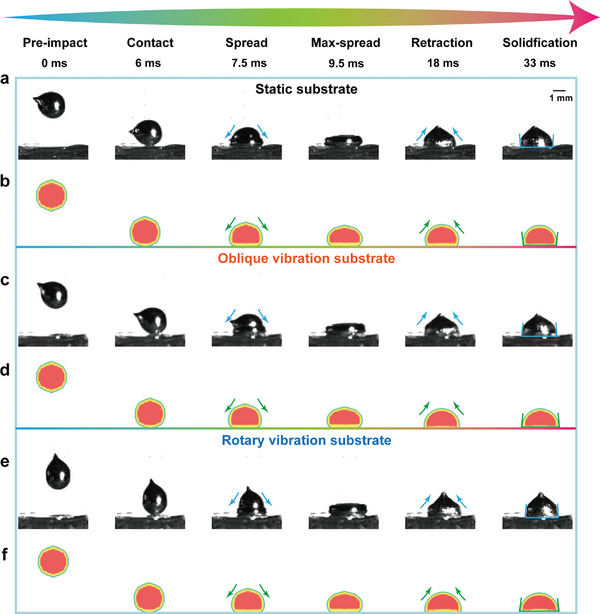
Comparison of deposition behaviors on static and vibrating substrates. a, b) Selected snapshots captured by high‐speed camera and simulation showing the metal droplet impacting on the static substrate, where the metal droplet exhibits a nonwetting state. c, d) Selected snapshots captured by high‐speed camera and simulation showing the metal droplet impacting on the vibration substrate with oblique trajectory, where the metal droplet exhibits a wetting state. e, f) Selected snapshots captured by high‐speed camera and simulation showing the metal droplet impacting on the vibration substrate with a circular trajectory, where the metal droplet exhibits a wetting state.

**Figure** **4** illustrates the key frames of the spreading process. The initial temperature and deposition distance for all experiments conducted in this work are set at 548 K and 10 mm, respectively. The experimental results indicate that the dynamics of metal droplets can be classified into four distinct stages: the rapid spreading stage, the maximum spreading stage, the retraction stage, and the oscillatory solidification stage. The time intervals between adjacent characteristic moments are calculated based on the number of frames. As shown in Figure 4a, when metal droplets impact a static glass substrate, a series of dynamic processes occur. Ultimately, the final contact angle between the solidified metal droplets and the glass substrate exceeds 90°, indicating a non‐wetting state. This observation aligns with the intrinsic non‐wetting properties of the metal droplets in relation to the glass substrate. The theoretical analysis of energy evolution is presented in Note S3, Supporting Information. Based on the dynamic processes obtained from experiments, the energy evolution at different stages can be summarized as follows: during the initial spreading phase on the static substrate, the metal droplet is primarily driven by its own kinetic energy. Once the metal droplet reaches its maximum spreading state, it begins to retract. During the entire retraction phase, surface energy becomes the dominant factor influencing droplet retraction. In this phase, the metal droplets experience both oscillation solidification and static solidification, leading to the formation of metal bumps. Ultimately, the completely solidified metal droplet exhibits a non‐wetting state with the substrate.

When the metal droplets impact the 2D ultrasonic vibration substrate, the dynamic spreading process initially resembles the state of the stationary substrate upon reaching maximum spreading. However, the static contact angle after solidification is measured to be less than 90°, indicating a wetting state after the droplets retract, in which the droplets ultimately solidify, as shown in Figure 4c,e. From the perspective of energy evolution, when the impacting droplet deposits on the vibrating substrate, additional ultrasonic energy is continuously injected into the deposition process. The influence of ultrasonic energy on droplet formation can be assessed by comparing the differences observed at characteristic times. The experimental results indicate that the metal droplet initially spreads due to its inherent kinetic energy. The primary effect of ultrasound on the spreading process results in an increase in the maximum spreading diameter. Once the metal droplet reaches its maximum spreading state, it begins to retract. During the entire retraction phase, surface energy and ultrasonic energy are the dominant factors influencing droplet retraction. Surface energy promotes the retraction of the metal droplet, while ultrasonic energy counteracts this effect, resisting the retraction of the droplet. In this state, the metal droplets experience both oscillation solidification and static solidification, leading to the formation of metal bumps. Ultimately, the completely solidified metal droplet exhibits a wetting state with the substrate. In short, the application of ultrasonic energy promotes the spreading behavior of metal droplets while simultaneously inhibiting their retraction, thereby creating a clear distinction between wetting and non‐wetting states.

Additionally, a fluid‐solid coupling model for the deposition of impacting metal droplets was created utilizing the finite element method. To enable bidirectional energy transfer between the metal droplets and the substrate, the surface of the deposition substrate was designated as a coupling surface, and the process of droplet deposition was simulated. Considering the physical characteristics of molten metal, molten liquid tin is categorized as a standard Newtonian fluid. By employing the basic principles of mass and energy conservation, we implement the volume of fluid (VOF) method to construct a model pertaining to the deposition of metal droplets. The keyframes from the simulation demonstrate the dynamic deposition of metal droplets on both static and vibrating substrates, as depicted in Figure 4b,d,f. The experimental results, along with the simulation results, confirm that the 2D ultrasound‐assisted metal droplet deposition technique can improve the wettability of metal droplets in the non‐wetting system, promoting the shift from a non‐wetting state to a wetting state. (Movies S3 and S4, Supporting Information)

**Figure 5:**
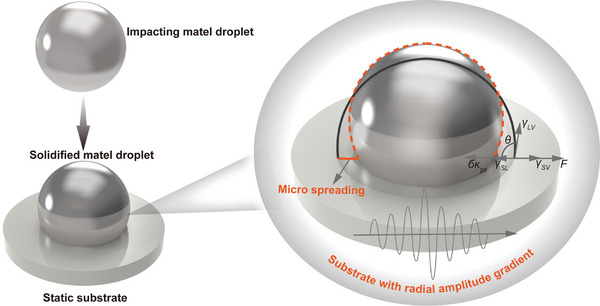
Mechanical diagram of microscale spreading phenomena.

From the perspective of mechanical analysis on the three‐phase contact line, in a non‐wetting system without an ultrasonic field, the three‐phase interface of the static droplet is influenced by the surface tensions at the gas‐liquid, liquid‐solid, and gas‐solid interfaces. Under these conditions, the forces are balanced, resulting in a net force of zero, and the wetting angle exceeds 90°. Consequently, spontaneous spreading behavior on the non‐wetting substrate is not observed. In contrast, the forces acting at the three‐phase interface are altered by the average additional ultrasonic force in the spreading direction when a specific intensity of ultrasonic field is applied. Besides, the initial impact also influences the spreading behavior of metal droplets. As a result, the interface forces are no longer in equilibrium. When the average additional ultrasonic force exceeds the combined effects of surface tensions, the metal droplets exhibit microscale spreading behavior under forced conditions, as shown in **Figure** **5**. The detailed mechanical analysis of microscale spreading phenomena is presented in Note S4, Supporting Information.

### 2.5 Wettability and Adhesive Strength of Solidified Metal Droplet

**Figure 6:**
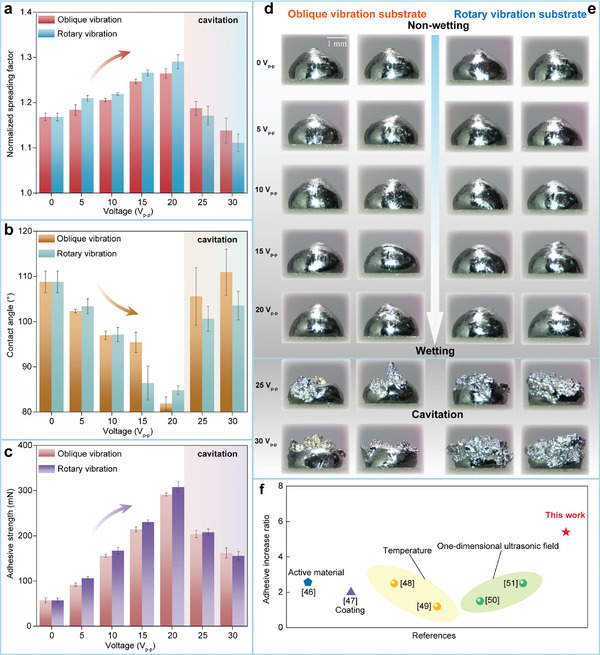
Wettability and adhesive strength of the solidified metal droplets. a) Normalized spreading factor of the solidified metal droplets versus the exciting voltage. b) Contact angle of the solidified metal droplets versus the exciting voltage. c) Adhesive strength versus the exciting voltage. d, e) The optical images of the solidified metal droplets under the treatment of the 2D ultrasonic field. f) Comparison of this work with other strategies for enhancing adhesion strength.

The influence of the 2D ultrasonic fields on the wettability and adhesive strength of the metal droplets was investigated through droplet deposition and solidification experiments. We analyzed the microstructural morphology of completely solidified tin droplets. The actual wetting behavior was characterized by measuring the maximum spreading diameter and the wetting angle between the metal droplet and the substrate. We examined the morphology of solidified metal droplets on three different types of substrates. In the experiment, the frequency applied to the 2D ultrasonic vibration device was set at 25.84 kHz, and the voltage ranged from 0 to 30 V_p‐p_. **Figure** **6**a illustrates the relationship between the spreading diameter of metal droplets and the applied voltage. The experimental results indicate that the normalized spreading factor of the solidified metal droplets on the static substrate is measured at 1.17. As the intensity of vibration increases, the spreading diameter of the metal droplets on the vibrating substrate also increases. The maximum recorded normalized spreading factor is 1.29 when the applied voltage reaches 20 V_p‐p_. Notably, the rotary vibrating substrate facilitates the spreading of metal droplets more effectively than the oblique vibrating substrate under the same vibration intensity. This observation suggests that when the substrate undergoes circular vibrations, the additional ultrasonic energy transmitted within the metal droplet is greater due to its coupling with the metal droplet. Figure 6b depicts the relationship between the contact angle and the applied voltage. The experimental results reveal that the contact angle of solidified metal droplets decreases as vibration intensity increases in the non‐wetting system. Specifically, within the range of 0 to 20 V_p‐p_, the average contact angle decreases from 108.8° to 81.9°, indicating a transition of metal droplets from a non‐wetting state to a wetting state. Figure 6d,e present optical images of the solidified metal droplets. The observed trends in spreading diameter and contact angle demonstrate that the application of 2D ultrasonic fields facilitates the transition of molten metal droplets from a non‐wetting state to a wetting state. Consequently, the wettability and morphology of solidified metal droplets can be effectively controlled by regulating ultrasonic intensity.

When the voltage reaches 25 V_p‐p_, the spreading and solidification process of metal droplets is accompanied by acoustic cavitation, which results in the breaking and detachment of the oxide film on the surface of the metal droplets. Once the metal droplets solidify, they no longer retain their original form as complete metal bumps. The experimental results indicate that acoustic cavitation can still occur in metal droplets in a semi‐solidified state, facilitating the removal of the oxide film. Furthermore, the severity of the acoustic cavitation phenomenon increases with greater vibration intensity. The microstructure of the metal droplets that undergo acoustic cavitation exhibits a non‐wetting state after solidification.

The adhesive strength is characterized by measuring the adhesion tangential force between the metal droplet and the substrate. The testing system is presented in Figure S8 (Supporting Information). Figure 6c illustrates the relationship between the adhesive strength and the applied voltage. The experimental results demonstrate that the 2D ultrasonic strategy can markedly enhance the adhesive strength. Specifically, under the applied voltage of 20 V_p‐p_, the adhesive strength is enhanced by 5.4 times compared to results obtained from deposition on the static substrate. The substantial increase in adhesive strength is primarily attributed to two key factors: the increase in the spreading diameter and the refinement of the contact region. Furthermore, Figure 6f highlights a comparison between methods observed in previous studies and the strategy proposed in this work. The adhesion increase ratio is employed to illustrate the extent of improvement in adhesive strength. Various existing methods for enhancing adhesive strength include incorporating active materials,^[^ ^46^ ^]^ utilizing coatings,^[^ ^47^ ^]^ managing temperature control,^[^ ^48^, ^49^ ^]^ and applying assistance through 1D ultrasonic field.^[^ ^50^, ^51^ ^]^ The innovative 2D ultrasonic strategy presented in this work offers a pronounced enhancement in adhesive strength between metal droplets and a nonwetting substrate. In conclusion, the comparison indicates that the proposed 2D ultrasonic strategy has significant potential to broaden the application scope of metal droplets within the realm of additive manufacturing.

## 3 Conclusion

In this work, a 2D ultrasonic strategy was proposed to enhance the wettability and adhesive strength of metal droplets on non‐wetting substrate, aiming to overcome the challenges posed by the high temperature, high viscosity, and large surface tension of metal droplets deposition on non‐wetting substrates. A 2D ultrasonic vibration device was designed utilizing orthogonal second‐order longitudinal vibration modes as the working modes. The 2D ultrasonic field was established based on the ultrasonic vibration at the deposition substrate. Furthermore, a vibration deposition substrate with radial vibration amplitude gradient was constructed based on the 2D ultrasonic field. The radial amplitude gradient could result in a spreading force directed from the center to the edge of the metal droplets, thereby enhancing the wettability and adhesive strength of impacting metal droplets ejected by a piezoelectric micro‐jet device. By adjusting the phase difference of the excitation signals, the 2D vibration trajectories were synthesized, enabling both oblique vibration and circular vibration. The vibration amplitude distributions and trajectories of the deposition substrate were verified through transient simulations utilizing the finite element method.

Additionally, the fluid flow mechanism for the metal droplets on the ultrasonic vibration substrate was simulated based on the VOF method. The prototype was fabricated, and a series of experiments were conducted with metal droplet deposition on the glass substrate. The dynamic behaviors of metal droplets on the static substrate and the vibrating substrate were obtained utilizing high‐speed video technology. Compared to the deposition on the stationary substrate, the application of 2D ultrasonic fields resulted in 10.3% improvement of the spreading diameter, while the corresponding contact angle was decreased by 24.7%, enabling the transition of metal droplets from a nonwetting state to a wetting state within the non‐wetting system. Additionally, the adhesive strength was enhanced by 5.4 times compared to deposition on the static substrate. The wettability and morphology of solidified metal droplets could be effectively regulated by adjusting ultrasonic intensity. In summary, the feasibility and effectiveness of the proposed 2D ultrasonic‐assisted metal droplet deposition strategy were verified through simulations and experiments. We anticipate that this method will broaden the application of metal droplets in surface coatings, additive manufacturing, and printed circuits on non‐wetting substrates. In the future, the investigation of multi‐dimensional ultrasonic field‐assisted metal droplet deposition on flexible substrates is a valuable area, which is of great significance for expanding the application fields.

## 4 Experimental Section

### Power Device

For the piezoelectric micro‐jet device, a typical trapezoidal pulse signal was generated and amplified via a waveform generator (Rigol, DG4162) and a power amplifier (Aigtek, ATA4052). The temperature of the molten metal was inferred with a K‐type thermocouple (Kaipusen, TTK30SLE). For the 2D ultrasonic vibration device, two sinusoidal excitation signals were applied via the ultrasonic power (QD‐8D), and a controllable substrate working at static and vibration modes with different phases and amplitudes was obtained.

### Characterizations

A high‐speed image acquisition system (Photron, Mini UX) was used to capture the dynamics of the metal droplets. The images were analyzed by the software Photron Fastcam Viewer. The optical images and size parameters of the solidified metal droplets were examined utilizing an optical stereo microscope (Supereyes, B013). The test conditions were maintained in a completely steady state. A pressure sensor (GJBHX‐III) was utilized to measure the adhesive strength. Note S5 (Supporting Information) shows the testing method.

### Materials and Fabrication

The 2D ultrasonic vibration device comprised four longitudinal ultrasonic transducers. Each driving unit consisted of a horn (2A12 duralumin), an end cap (304 stainless steel), nine electrodes (Copper), eight piezoelectric ceramics (PZT‐4), and a M10 preload bolt (42CrMo alloy steel). Note S6 (Supporting Information) shows the assembly process of the prototype.

### Numerical Methods

The finite element modeling of the 2D ultrasonic vibration device was performed using ANSYS APDL language. The material parameter settings for each component were presented in Note S7 (Supporting Information). The numerical calculations of the impacting metal droplets were conducted in ANSYS Fluent based on the VOF method, Note S8 (Supporting Information). The parameters of tin are listed in Table S1 (Supporting Information).

### Statistical Analysis

Data were expressed as mean ± SD (Standard Deviation). The sample size for each statistical analysis is *n *= 3. Statistical analysis of the data was performed using Origin 2022.

## Conflict of Interest

The authors declare no conflict of interest.

## Supporting information



Supporting Information

Supporting Information

Supporting Information

Supporting Information

Supporting Information

## Data Availability

The data that support the findings of this study are available from the corresponding author upon reasonable request.
