# Supplementary material for: Wetting and Spreading Behaviors of Impacting Metal Droplet Regulated by 2D Ultrasonic Field
Source: Adv Sci (Weinh). 2025 Jan 30;12(11):2415138. doi: 10.1002/advs.202415138 (PMC11923880; doi:10.1002/advs.202415138)
Supplement: Supplementary file 1 — Supporting Information [file ADVS-12-2415138-s004.docx]

Supporting Information

**Wetting and Spreading Behaviors of Impacting Metal Droplet Regulated by Two-Dimensional Ultrasonic Field**

*Yuzhu Zhao, Shijing Zhang, Jing Li, Jie Deng*, Yingxiang Liu**

Email: dengjie21@hit.edu.cn (J.D.), liuyingxiang868@hit.edu.cn (Y.L.).

**The PDF file includes:**

Supplementary Note S1 to S8

Figure S1 to S8

Table S1

**Other Supporting Information includes the following:**

Movie S1 to S4

Supplementary Note

**Note S1. Generation of the two-dimensional orthogonal ultrasonic field**

By exciting the piezoelectric ceramics, the second-order longitudinal vibration modes of the device are induced through the inverse piezoelectric effect. The deposition surface concurrently generates high-frequency harmonic vibrations along the X axis and Y axis based on the fundamental working principle of longitudinal vibration mode recombination. This results in the formation of a two-dimensional orthogonal ultrasonic field on the deposition surface.

We label the center point of the deposition surface as Q. By exciting all the driving units simultaneously with two AC signals of the same frequency, the vibration displacement of Q along each direction can be described by (1) and (2). The two-dimensional vibration trajectory expression is obtained by triangular transformation and elimination, as described by (3).

$d_{\text{X}}=D_{\text{X}}\cos(2\pi ft+\alpha)$ (1)

$d_{\text{Y}}=D_{\text{Y}}\cos(2\pi ft+\beta)$ (2)

$\left(\frac{d_{\text{X}}}{D_{\text{X}}}\right)^{2}+\left(\frac{d_{\text{Y}}}{D_{\text{Y}}}\right)^{2}-\frac{2\cos(\alpha-\beta)}{D_{\text{X}}D_{\text{Y}}}d_{\text{X}}d_{\text{Y}}=\sin^{2}(\alpha-\beta)$ (3)

where *d*_X_, *d*_Y_ represent the vibration displacements, *D*_X_, *D*_Y_ are the maximum vibration amplitudes, *f* is the working frequency, and *α*, *β* are the initial phases of the excitation signals.

The influence of phase difference: From an electrical perspective, two-dimensional vibration trajectory synthesis is accomplished by applying excitation signals with different phases at a specific frequency. Altering the phase difference between the two phases of excitation signals leads to changes in the synthetic direction and the fullness of the vibration trajectory. Consequently, this affects the contact state of the coupling between the metal droplet and the substrate.

$S_{33}=d_{33}E_{33}$ (4)

where *S*_33_ represent the strain in the thickness direction of PZT, *d*_33_ is the piezoelectric constants in the thickness direction of PZT, *E*_3_ is electric field strength in the thickness direction of PZT.

The influence of voltage: The deposition behaviors are directly influenced by the vibration amplitude of the substrate. The piezoelectric constitutive equation can be described by (4). The deformation of PZT is proportional to the electric field strength. Increasing the excitation voltage, in principle, enhances the vibration amplitude of the substrate.

**Note S2. Testing method of the vibration characteristics**

The Doppler laser vibration measuring system (Model: PSV-400, Polytec, Germany) shown in Figure S3 was utilized for vibration characterization tests. The vibration characterization tests were conducted to determine the optimal excitation frequency of the vibration mode. These tests involved obtaining the actual vibration pattern and the vibration velocity at each point on the surface of the base. The system includes the laser head, data management system and controller. The laser head was used to emit and receive laser light. The data manager was used to process data about the vibration of the measured object. A controller and power amplifier (ATA-4051, Aigtek, China) were used to transmit the beep signal. The laser was vertically irradiated into the end face of the end caps. The vibration response curves and actual vibration patterns of the required vibration modes were obtained by sweeping. The chirp signals with amplitude of 10 Vp-p and frequency range of 10~50 kHz was applied to the piezoelectric ceramics along X axis and Y axis, respectively.

**Note S3. Theoretic analysis on energy evolution.**

As shown in **Figure S7**, in the context of impacting droplet deposition on the static substrate, the energy evolution can be categorized into three distinct stages^[1]^: the initial moment of collision with the substrate, the attainment of the maximum spreading diameter, and the subsequent retraction until solidification.

The kinetic energy, surface energy, and gravitational potential energy at the initial moment of metal droplet collision with the substrate can be described as:

$E_{K1}\text{=}\frac{\pi}{12}\rho D_{0}^{3}V_{0}^{2}$ (5)

$E_{S1}\text{=}\pi D_{0}^{2}\sigma$ (6)

$E_{P1}\text{=}\frac{\pi}{12}\rho D_{0}^{4}g$ (7)

Then, the droplet gradually spread until reaching the maximum spreading diameter. The surface energy is:

$E_{S2}\text{=}\frac{\pi}{4}D_{a}^{2}\sigma(1-\cos\theta_{a})$ (8)

where *θ_a_* is the advancing solid-liquid contact angle.

The work caused by adhesion force form the initial moment to the maximum spreading can be described as:

$W\text{=}\frac{\pi}{3\sqrt{\mathrm{Re}}}\rho D_{0}D_{a}^{2}V_{0}^{2}$ (9)

where Re is the Reynolds number (Re=*ρVD*/*μ*).

The kinetic energy during the spreading process can be calculated as:

$\Delta E_{K}\text{=}\frac{\pi}{8}\rho sD_{a}^{2}V^{2}$ (10)

where *s* is the thickness of the film layer and *D_a_* is the diameter during spreading.

The change in the gravitational potential energy can be described as:

$\Delta E_{P}\text{=}\pi\rho g\int_{0}^{D_{0}} yx^{2}dz$ (11)

The energy balanced equation during the spreading process can be described as:

$E_{S1}+E_{K1}+E_{P1}=E_{S2}+\Delta E_{K}+\Delta E_{P}\text{+W}$ (12)

The kinetic energy is zero when the droplet reaches the maximum spreading diameter. After the droplet reaches the maximum diameter, the surface tension will cause the droplet to retract until the droplet could completely solidify, which the system reaches steady state. The surface energy and potential energy during retracting can be described as:

$E_{S3}\text{=}\frac{\pi}{4}D_{r}^{2}\sigma(1-\cos\theta_{r})$ (13)

$E_{P3}\text{=}\frac{\pi}{4}\rho gD_{r}^{2}h^{2}$ (14)

where *D_a_* is the diameter during retracting, *θ_r_* is the contact angle during retracting, h is the height of droplet.

**Note S4. Mechanical analysis of microscale spreading phenomena.**

Micro wetting mechanism of impacting droplet on static substrate: The stable contact angle is employed to characterize the wettability between the solid and liquid phases. At the macro scale, this contact angle is primarily influenced by the interfacial tensions of the liquid-vapor, solid-vapor, and solid-liquid phases.^[2,3]^ The Young's equation is utilized to describe the equilibrium among these three interfacial tensions. The classical formulation of Young's equation is expressed as follows:

$\gamma_{LV}\text{cos}\theta_{\infty}+\gamma_{SL}-\gamma_{SV}\text{=}0$ (15)

where *γ_LV_*, *γ_SV_*, and *γ_SL_* are liquid-vapor, solid-vapor, and solid-liquid interface tensions. *θ_∞_* is the equilibrium contact angle.

When a droplet impacts a solid surface, it has been demonstrated that the dynamic contact angle and spreading diameter undergo damping oscillations over time.^[4]^ Consequently, the three-phase contact line (TPCL) of the impacting droplet becomes unbalanced during the dynamic process on the substrate.

To analyze the variations in the dynamic contact angle of the droplet, line tension is introduced in accordance with the classical Young’s equation. The interfacial tensions near the TPCL will be rebalanced under the influence of line tension. Therefore, based on the equilibrium condition of forces, the first-order modified Young’s equation is formulated as follows:

$\gamma_{LV}\text{cos}\theta+\gamma_{SL}+\sigma\kappa_{gs}-\gamma_{SV}\text{=}0$ (16)

where *θ* and *κ_gs_* are the dynamic contact angle and geodesic curvature of the TPCL. *σ* is the term used as apparent line tension.

The line tension is numerically equivalent to the free energy per unit length of the TPCL, with its direction oriented towards the center of the circle. This orientation seeks to minimize the length of the TPCL. We assume that the spreading processes of the impacting droplet are axisymmetric. Consequently, the geodesic curvature is expressed as follows:

$\kappa_{gs}\text{=}\frac{1}{r}$ (17)

where *r* is the contact radius of the droplet.

The dynamic contact angle for impacting droplet can be described as:

$\text{cos}\theta\text{=cos}\theta_{\infty}-\frac{\sigma}{\gamma_{LV}\cdot r}$ (18)

Eq. (18) indicates that the contact angle will vary with the radius of the TPCL before the system reach steady state. For a given solid-liquid system, cos*θ*_∞_ is a constant value.

Micro wetting mechanism of impacting droplet on vibrating substrate: In this work, we construct a two-dimensional ultrasonic substrate featuring a radial amplitude gradient. The vibration amplitude is maximal at the center and decreases towards the edges. Consequently, this radial amplitude gradient generates a spreading force directed from the center to the edge of the droplet when the vibrating substrate interacts with the metal droplet. Upon applying ultrasonic vibration to the substrate, an additional tension is induced, which can push the TPCL outward. Furthermore, a viscous momentum transfer layer, induced by the ultrasonic shear waves, is established at the solid-liquid interface.^[5,6]^ This momentum transfer layer interacts with the droplet surface near the TPCL, leading to a dynamic TPCL that ultimately reaches a new equilibrium position under ultrasonic action. The second-order correction of Young’s equation is formulated as:

$\gamma_{LV}\text{cos}\theta+\gamma_{SL}+\sigma\kappa_{gs}-\gamma_{SV}-F\text{=}0$ (19)

where *F* is the additional acoustic tension induced by ultrasonic vibration.

The radical acoustic tension per unit length along the contact line^[7]^ can be calculated as:

$F\text{=}\frac{\rho}{32\beta^{-1}}(U_{c}r)^{2}\cos^{2}\theta$ (20)

$U_{c}\text{=}2\pi f\xi_{0}$ (21)

where *U_c_* is the streaming velocity and *ξ*_0_ is the surface vibration half-amplitude.

The interfacial tension created by the viscous momentum transfer layer is located in the submicron region, and the thickness of this region can be expressed as:

$\beta^{-1}\text{=}\sqrt{\frac{\mu}{\pi f\rho}}$ (22)

where *μ* and *ρ* are the viscous and density of the droplet, *f* is the vibration frequency of the substrate.

Substituting Eq. (20) into Eq. (19) yields the following equation:

$\gamma_{LV}\text{cos}\theta+\gamma_{SL}+\sigma\kappa_{gs}-\gamma_{SV}-\frac{\rho}{32\beta^{-1}}(U_{c}r)^{2}\cos^{2}\theta\text{=}0$ (23)

Therefore, the dynamic contact angle can be described with the following equation:

$\text{cos}\theta-\text{cos}\theta_{\infty}=We\frac{r\cos^{2}\theta}{64\sqrt{\mu/\pi f\rho}}-\frac{\sigma}{\gamma_{LV}r}$ (24)

where *We* is used to present Weber number which defines the ratio of inertia force to surface tension.

The terms on the left side of Eq. (24) are defined by the combination of the interfacial tensions. The terms on the right side of Eq. (24) are defined by the force of ultrasound and the line tension of the impacting droplet. In the non-wetting system, when the average additional ultrasonic force surpasses the combined effects of the interfacial tensions, the metal droplets demonstrate an outward spreading behavior under forced conditions. Consequently, the spreading behavior can be induced by the ultrasonic field.

The liquid-vapor interface and the TPCL of the spreading droplet also exhibit small vibrations with the injection of ultrasonic vibration energy. In addition, the capillary waves on the surface of the droplet are created along with these vibrations. The interfacial roughness caused by ultrasound will lead to a change in the free energy at TPCL.^[2]^ Then, the change in the free energy per unit length can be described as:

$C_{W}\gamma_{LV}\text{(cos}\theta-\text{cos}\theta_{\infty})=\text{d}G/\mathrm{dr}$ (25)

where *C_W_* is used to define the ratio of the waveform length *A_L_* of capillary wave to its wavelength.

In the non-wetting system, it is assumed that the left and right sides of the droplet on the horizontal vibration substrate are symmetrical. The free energy on both sides of the droplet is approximately equal. Therefore, the TPCL of the droplet symmetrically spreads in micro along the substrate surface.

In summary, the radial amplitude gradient results in a spreading force directed from the center to the edge of the droplet when the horizontal vibration substrate interacts with the metal droplet. The momentum transfer layer and the capillary waves induced by the additional ultrasonic force are the origin factors of the influence on wettability.

**Note S5. Testing method of the adhesive strength.**

The adhesion tangential force is measured to quantify the adhesion force between the solidified Sn droplet and the substrate. As shown in Fig. S8, the adhesion tangential force measurement system consists of a 3D motion platform, a pressure sensor (GJBHX-III), a scraper, a transmitter (GJ-4057A), and a digital input module (NI-9215). First, the scraper fixed on the pressure sensor, is adjusted right below the Sn droplet on a vibration substrate. Then the measurement program based on the LabVIEW is open while the scraper moves up towards the droplet. The data acquisition process is completed when the droplet is separated from the substrate. During the process, the adhesion tangential force is measured and amplified by the pressure sensor and the transmitter, and then the voltage signal is analyzed and recorded by the digital input module. The final data storage and waveform display are performed by the LabVIEW program on a computer.

**Note S6. The assembly process of the prototype.**

The overall structure of the two-dimensional ultrasonic vibration deposition device includes piezoelectric ceramics, electrode sheets, horns, end caps, and bolts. The assembly process of the prototype is as follows:

Step 1: Clean the surfaces of each component with ethanol.

Step 2: Prepare eight monopolar piezoelectric ceramics, and nine electrodes.

Step 3: Put electrodes and ceramics alternately with the epoxy resin adhesive.

Step 4: Finish the assembly of one driving units and weld the same electrode pins together.

Step 5: Repeat the above steps to get another three driving units.

Step 6: Assemble the horns, the driving units, and the end covers with M10 preload bolts to get the prototype.

Step 7: Paste the glass substrate to the deposition surface with the epoxy resin adhesive.

After assembly, the volume of the prototype is measured as 186 × 186 × 35 mm^3^.

**Note S7. Material parameters of the two-dimensional ultrasonic vibration device**

The material parameter settings for each component of the two-dimensional ultrasonic vibration device are presented as follows: The PZT elements adopt PZT-4 (Lead Zirconate Titanate, a commercialized piezoelectric material with density of 7.6×10^3^ kg/m^3^, provided by Baoding Hongsheng acoustic electronic equipment Co., Ltd, China) with outer diameter of 30 mm, inner diameter of 12 mm, and thickness of 1 mm. The end cap is made of stainless steel 304, which has excellent anti-corrosion properties (Density of 7.93×10^3^ kg/m^3^, Young’s modulus of 1.94×10^11^ N/m^2^, and Poisson’s ratio of 0.31); the horns are assembled from aluminum (Density of 2.77×10^3^ kg/m^3^, Young’s modulus of 7.1×10^10^ N/m^2^ and Poisson’s ratio of 0.33). The metal parts are meshed by SOLID186 unit. The PZT elements are meshed by SOLID227 unit. The remaining physical parameters of the PZT-4 are obtained from the supplier as follows:

$\left[e\right]=\left[\begin{matrix} 0 & 0 & -2.4 \\ 0 & 0 & -2.4 \\ 0 & 0 & 17.3 \\ 0 & 0 & 0 \\ 0 & 12.95 & 0 \\ 12.95 & 0 & 0 \end{matrix}\right]\text{ C}/\text{m}^{2}$ (26)

$\left[\varepsilon\right]=\left[\begin{matrix} 765 & 0 & 0 \\ 0 & 765 & 0 \\ 0 & 0 & 640 \end{matrix}\right]\times10^{-11}\text{F/m}$ (27)

$\left[c^{E}\right]=\left[\begin{matrix} 14.3 & 7.85 & 7.85 & 0 & 0 & 0 \\ 7.85 & 14.3 & 7.85 & 0 & 0 & 0 \\ 7.85 & 7.85 & 11.5 & 0 & 0 & 0 \\ 0 & 0 & 0 & 2.45 & 0 & 0 \\ 0 & 0 & 0 & 0 & 2.6 & 0 \\ 0 & 0 & 0 & 0 & 0 & 2.6 \end{matrix}\right]\times10^{10}\text{N/m}^{2}$ (28)

where *e*, *ε* and *c^E^* were the piezoelectric stress constant matrix, the dielectric matrix at constant stress and the stiffness matrix at constant electric field, respectively.

**Note S8. Multi-physics numerical model validation.**

A fluid-structure interaction simulation model is established to simulate the spreading dynamics of the metal droplet on the surface with ultrasonic vibration using the commercial software ANSYS Fluent. The molten metal is a typical Newtonian fluid, and the flow is assumed to be laminar and incompressible, which is governed by the conservations of mass, momentum, and energy. Regarding the two phases, the primary one is set as air, while the secondary one is set as liquid tin.

References

[1] S. D. Aziz, S. Chandra, *Int. J. Heat Mass Transf.* **2000**, 43, 2841.

[2] B. Zhao, S. Luo, E. Bonaccurso, G. K. Auernhammer, X. Deng, Z. Li, L. Chen, *Phys. Rev. Lett.* **2019**, 123, 094501.

[3] J. Fan, J. De Coninck, H. Wu, F. Wang, *Phys. Rev. Lett.* **2020**, 124, 125502.

[4] Y. Feng, J. Liu, H. Li, X. Ma, P. Du, K. Li, Y. Liu, *Int. J. Heat Mass Transf.* **2022**, 192, 122902.

[5] X. Liu, Q. Jia, Y. Fu, T. Zheng, *Ultrason. Sonochem.* **2022**, 83, 105943.

[6] P. Kim, C. Duprat, S. S. H. Tsai, H. A. Stone, *Phys. Rev. Lett.* **2011**, 107, 034502.

[7] O. Manor, M. Dentry, J. R. Friend, L. Y. Yeo, *Soft Matter* **2011**, 7, 7976.


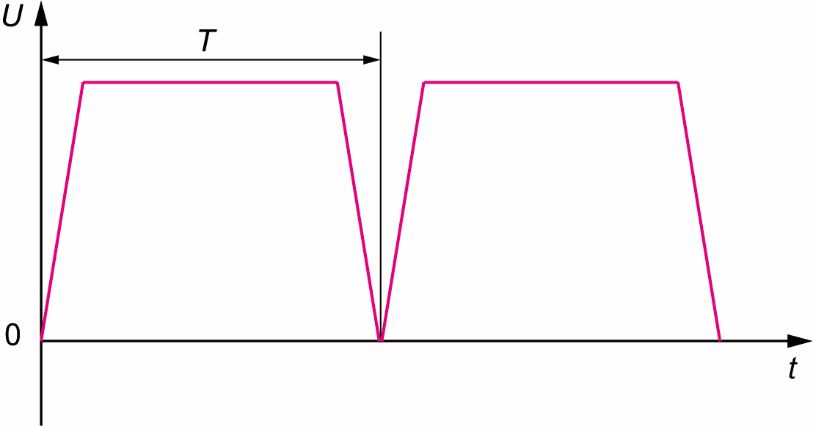


Figure S1. Excitation signal of the piezoelectric micro-jet device.


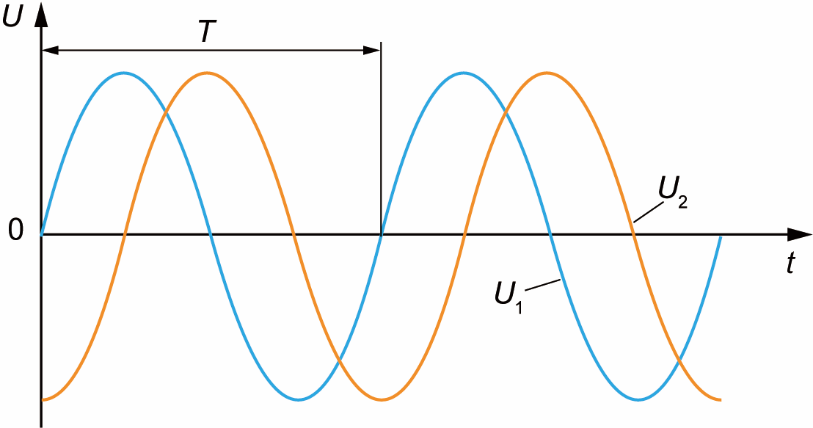


Figure S2. Excitation signals of the two-dimensional ultrasonic vibration device.


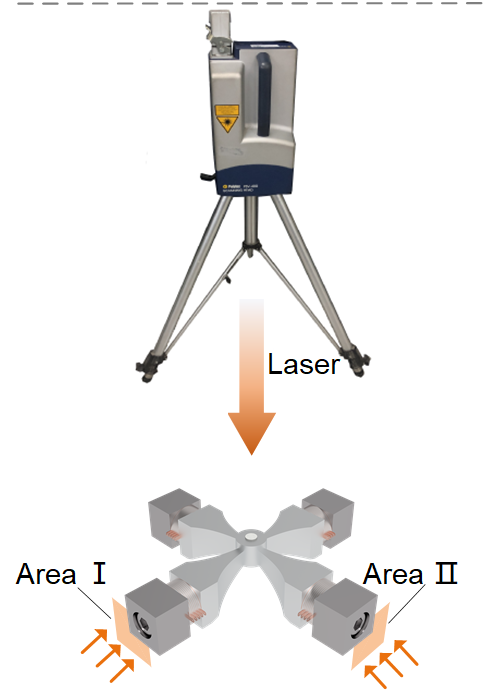


Figure S3. Configuration of the vibration testing systems.


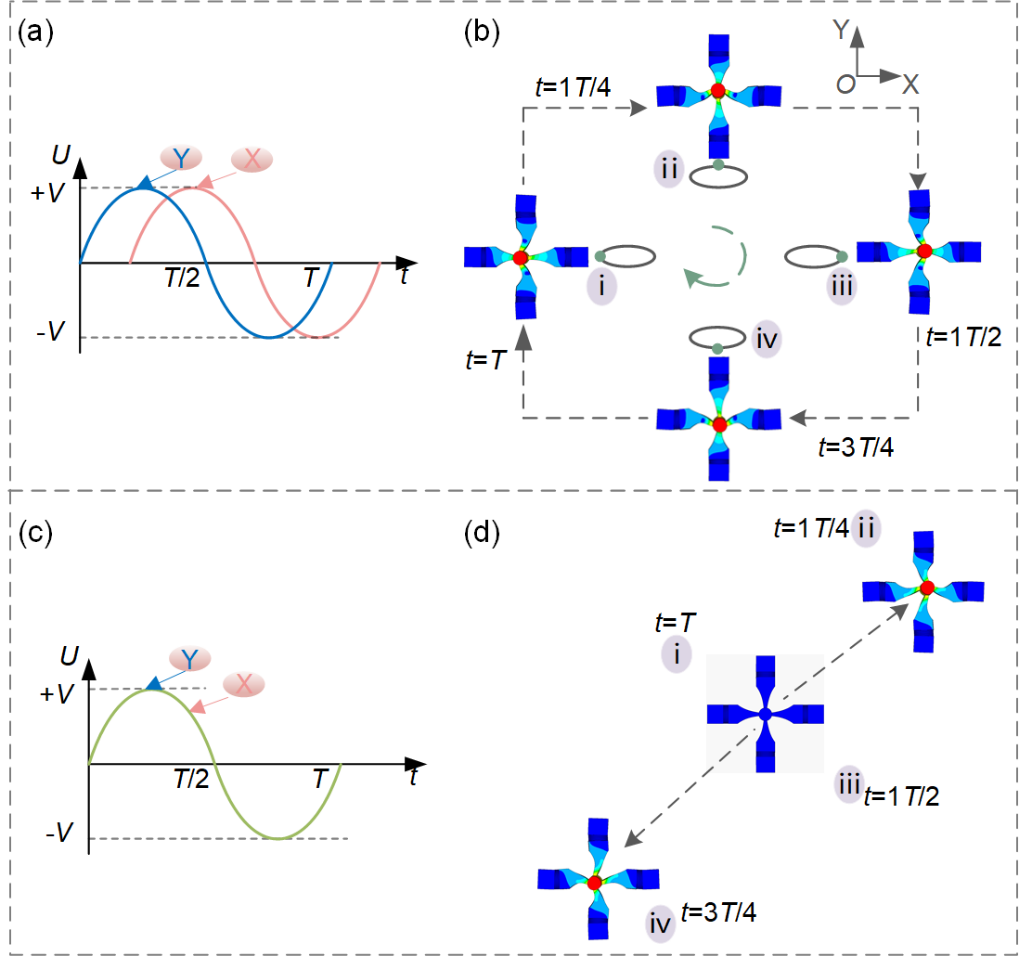


Figure S4. The transient simulation of the two-dimensional ultrasonic vibration device. a) AC signals. b) Circular vibration on four special moments. c) AC signals. d) Oblique vibration on four special moments.


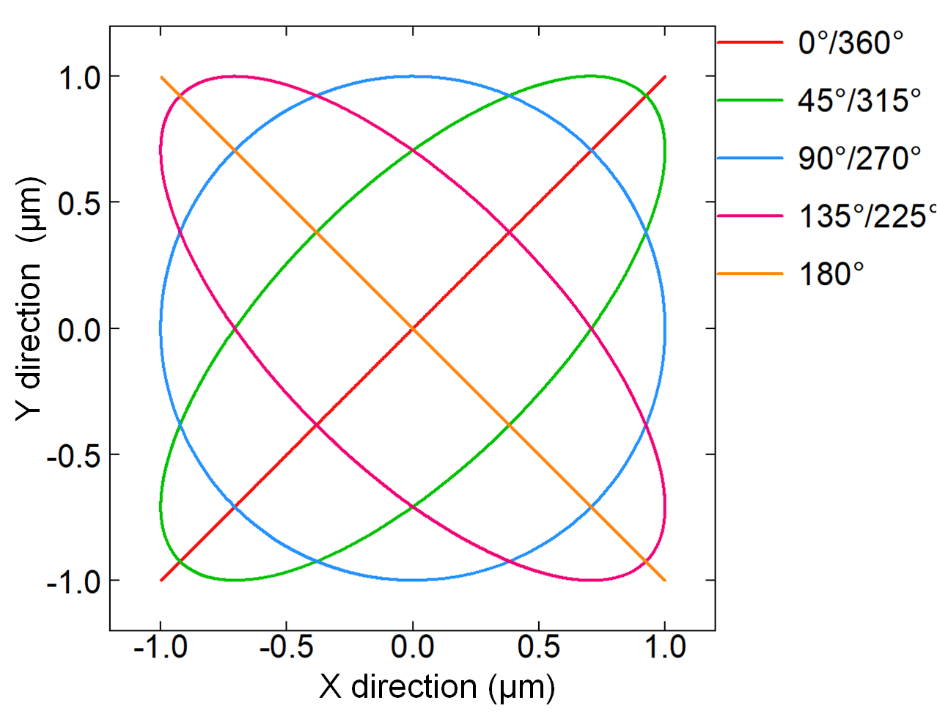


Figure S5. Vibration trajectories under different phase differences.


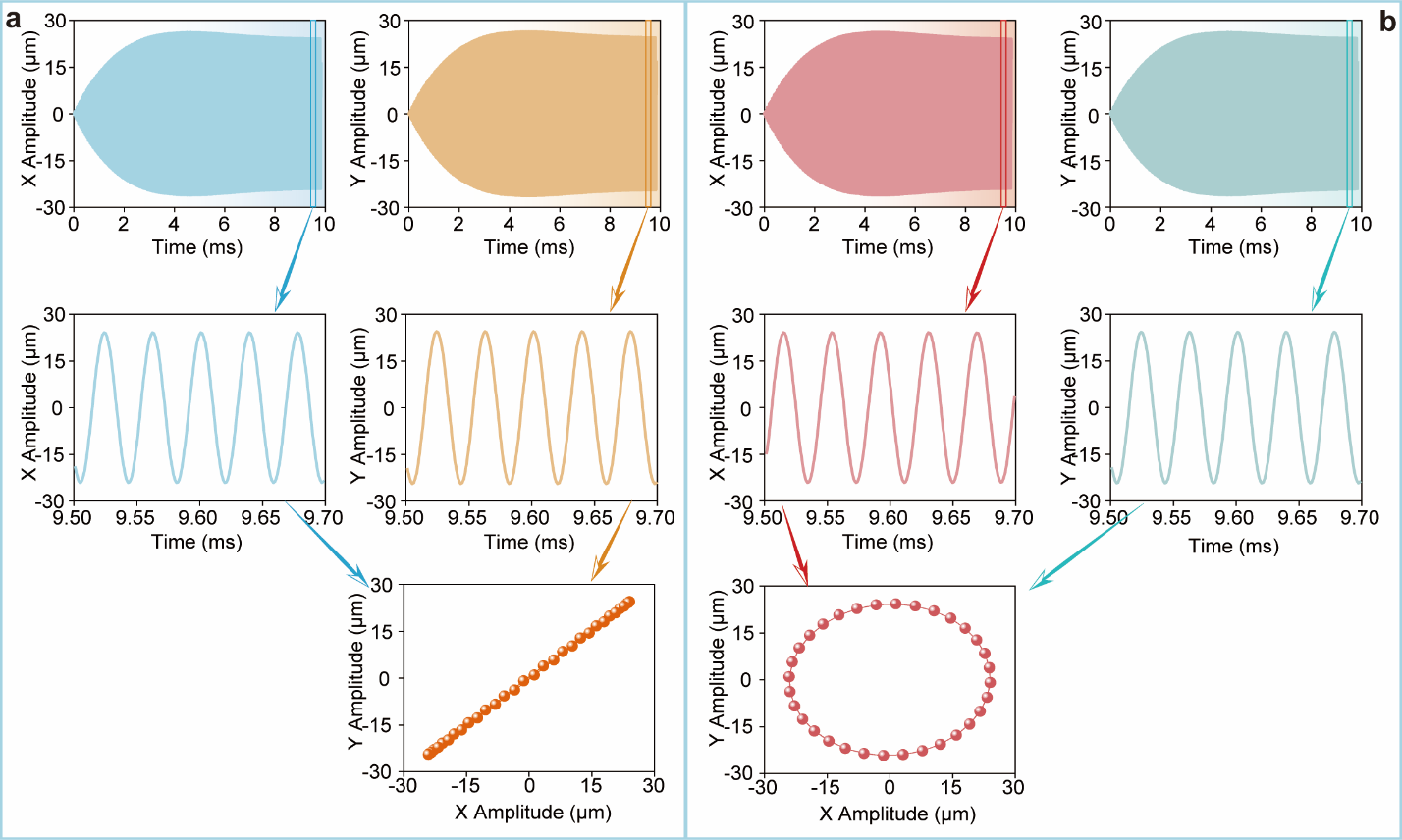


Figure S6. Vibration response and trajectories under the voltage of 200 V_p-p_. a) Oblique vibration. b) Circular vibration.


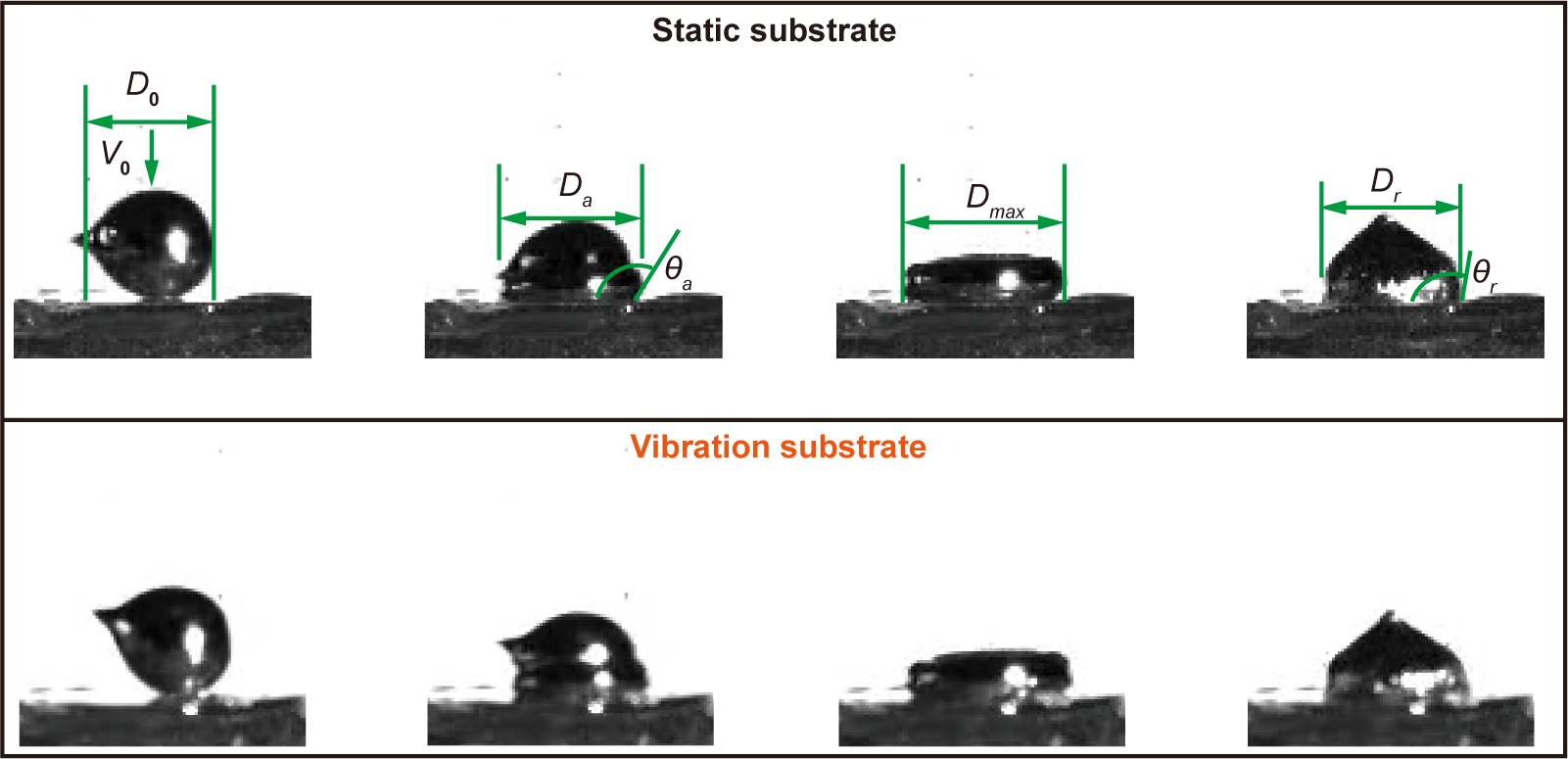


Figure S7. Energy evolution via droplet deposition behaviors.


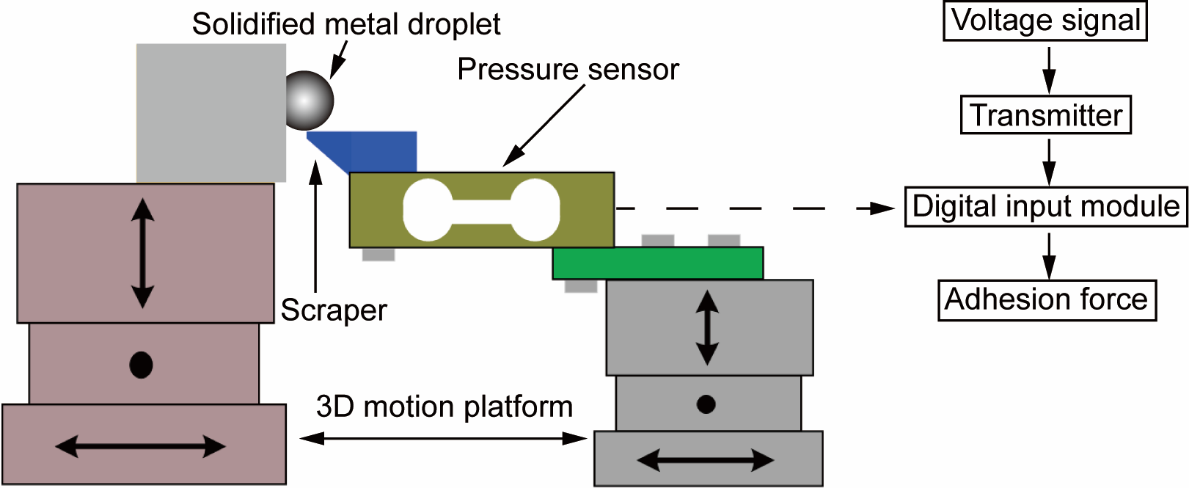


Figure S8. Configuration of the adhesive strength measurement systems.

**Table S1. Physical properties of tin.**

| Parameters | Values | Units |
| --- | --- | --- |
| Density | 7000 | kg·m^-3^ |
| Dynamic viscosity | 1×10^-3^ | Pa·s |
| Melting point | 498 | K |
| Specific heat | 220 | J·(kg·℃)^-1^ |
| Latent heat of fusion | 60.3 | J·g^-1^ |
| Thermal conductivity | 67 | W·(m·K)^-1^ |
| Surface tension | 0.57 | N·m^-1^ |

Movie S1.

Overall structure of the experiment setup.

Movie S2.

Generation of two-dimensional ultrasonic field.

Movie S3.

Simulation of metal droplet deposition behaviors.

Movie S4.

Experiment of metal droplet deposition behaviors.
